# Point of care parenchymal volume analyses to estimate split renal function and predict functional outcomes after radical nephrectomy

**DOI:** 10.1038/s41598-023-33236-6

**Published:** 2023-04-17

**Authors:** Nityam Rathi, Worapat Attawettayanon, Yosuke Yasuda, Kieran Lewis, Gustavo Roversi, Snehi Shah, Andrew Wood, Carlos Munoz-Lopez, Diego A. Palacios, Jianbo Li, Nour Abdallah, Jared P. Schober, Marshall Strother, Alexander Kutikov, Robert Uzzo, Christopher J. Weight, Mohamed Eltemamy, Venkatesh Krishnamurthi, Robert Abouassaly, Steven C. Campbell

**Affiliations:** 1 Glickman Urological and Kidney Institute, Cleveland Clinic, Cleveland, OH USA; 2 Division of Urology, Department of Surgery, Faculty of Medicine, Songklanagarind Hospital, Prince of Songkla University, Songkhla, Thailand; 3 Department of Urology, Tokyo Medical and Dental University, Tokyo, Japan; 4 Department of Quantitative Health Sciences, Cleveland Clinic, Cleveland, OH USA; 5 Department of Surgery, Division of Urologic Surgery, University of Nebraska Medical Center, Omaha, NE USA; 6 Department of Urology, Oregon Health Sciences University, Portland, OR USA; 7 Department of Urology, Fox Chase Cancer Center, Philadelphia, PA USA

**Keywords:** Urology, Kidney, Renal cancer, Surgical oncology

## Abstract

Accurate prediction of new baseline GFR (NBGFR) after radical nephrectomy (RN) can inform clinical management and patient counseling whenever RN is a strong consideration. Preoperative global GFR, split renal function (SRF), and renal functional compensation (RFC) are fundamentally important for the accurate prediction of NBGFR post-RN. While SRF has traditionally been obtained from nuclear renal scans (NRS), differential parenchymal volume analysis (PVA) via software analysis may be more accurate. A simplified approach to estimate parenchymal volumes and SRF based on length/width/height measurements (LWH) has also been proposed. We compare the accuracies of these three methods for determining SRF, and, by extension, predicting NBGFR after RN. All 235 renal cancer patients managed with RN (2006–2021) with available preoperative CT/MRI and NRS, and relevant functional data were analyzed. PVA was performed on CT/MRI using semi-automated software, and LWH measurements were obtained from CT/MRI images. RFC was presumed to be 25%, and thus: Predicted NBGFR = 1.25 × Global GFR_Pre-RN_ × SRF_Contralateral_. Predictive accuracies were assessed by mean squared error (MSE) and correlation coefficients (*r*). The *r* values for the LWH/NRS/software-derived PVA approaches were 0.72/0.71/0.86, respectively (*p* < 0.05). The PVA-based approach also had the most favorable MSE, which were 120/126/65, respectively (*p* < 0.05). Our data show that software-derived PVA provides more accurate and precise SRF estimations and predictions of NBGFR post-RN than NRS/LWH methods. Furthermore, the LWH approach is equivalent to NRS, precluding the need for NRS in most patients.

## Introduction

Radical nephrectomy (RN) and partial nephrectomy (PN) are the mainstays of treatment for localized renal cell carcinoma (RCC). While RN is often required for RCC tumors that demonstrate increased complexity and oncologic potential, the imminent risk of decline in new baseline glomerular filtration rate (NBGFR) relative to PN can be problematic^1^. Thus, accurate predictions of NBGFR after RN can have significant clinical implications for RCC management and patient counseling, particularly in cases where RN and PN offer unique advantages. Preoperative global GFR, split renal function (SRF), and postoperative renal functional compensation (RFC) in the contralateral kidney, also known as compensatory hypertrophy, are fundamentally important parameters for the accurate prediction of NBGFR after RN^2^. Recent studies have shown that a simplified model based on these three parameters provides more accurate predictions of NBGFR after RN than complex, multivariate algorithms developed from big data approaches^3^.

SRF has traditionally been obtained from nuclear renal scans (NRS), which rely on radiotracers, such as (99 m)Tc-DTPA and (99 m)Tc-MAG(3), to delineate renal anatomy, pathology, and physiology^4^. However, concerns regarding NRS include subjectivity when defining regions of interest and poor resolution of anatomical structures when compared to CT/MRI^5^. Recent studies suggest that software-derived differential parenchymal volume analysis (PVA) (FUJIFILM Medical Systems, USA) is more accurate for estimating SRF than NRS^6,7^. Briefly, the PVA software readily performs deep learning-based image analysis of cross-sectional CT or MRI scans to provide instant measurements of renal parenchymal and tumor volumes. The PVA software requires minimal human intervention, which reduces subjectivity of the approach.

A simplified approach to estimate parenchymal volumes and SRF based on linear length, width, and height measurements (LWH) has also been proposed^8,9^ This method incorporates average measurements of renal parenchymal length and thickness in different axes to provide an estimate of the relative parenchymal volumes. Of note, the linear measurements can be readily obtained from tools present in the CT/MRI user interface at point of care. In a recent, limited study of 60 patients, this method appeared to be equivalent to NRS in terms of predicting NBGFR after RN^8^.

In this study, we compare the accuracies of each of these three methods (NRS, software-derived PVA, and linear LWH Measurements) for estimating SRF, and, by extension, predicting NBGFR after RN in an expanded, independent cohort. We also discuss the clinical utility and practicalities of each of these approaches in terms of strengths, limitations, and efficiency when estimating SRF.

## Methods

### Ethics approval

All procedures involving human participants in the present study were conducted in accordance with the ethical standards of the institutional research committee and the 1964 Helsinki Declaration and its later amendments or comparable ethical standards. The study protocol was approved by the Cleveland Clinic Foundation Institutional Review Board approval (CCF IRB 20–836). Given the retrospective nature of the study, the need for informed consent was waived by the institutional review committee.

### Study population

**Figure 1:**
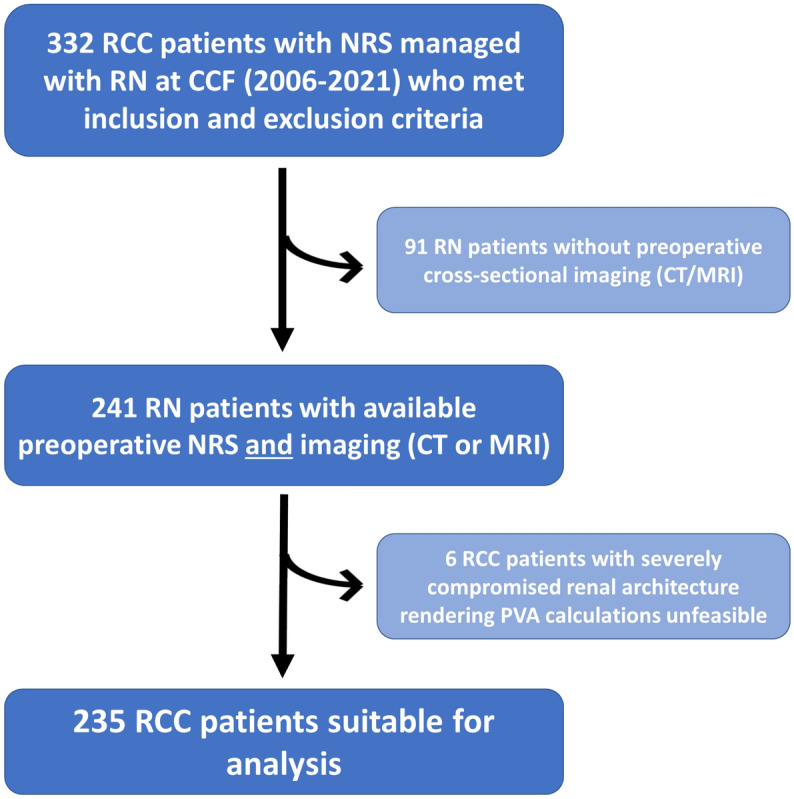
Inclusion and exclusion criteria for the final study cohort of 235 RCC patients managed with radical nephrectomy.

A total of 2240 RCC patients were managed with RN at the Cleveland Clinic from 2006–2021. Inclusion into the study required: (1) availability of preoperative (< 1 month pre-RN) and new baseline GFR (3–12 months post-RN) measures of renal function; (2) presence of a cancer-free contralateral kidney; (3) a preoperative eGFR > 15 ml/min/1.73 m^2^; and (4) available preoperative Tc-99 m MAG3 NRS with estimated SRF. This left 332 patients potentially eligible for analysis, of which 91 were excluded due to lack of preoperative cross-sectional imaging to allow for estimation of SRF by differential PVA (Fig. 1). Six patients were further excluded due to significantly compromised renal architecture that rendered PVA unfeasible, such as in the case of autosomal dominant polycystic kidney disease, culminating in a final cohort of 235 patients suitable for analysis (Fig. 1). Pertinent data on patient demographics, comorbidities, tumor characteristics, and renal function were collected.

Estimates of GFR in the preoperative and postoperative settings were based on Chronic Kidney Disease Epidemiology (CKD-EPI) collaboration formula^10,11^. NBGFR was defined as the final GFR obtained within 3–12 months in the postoperative period to allow for maximal recovery and functional compensation in the contralateral kidney.

### Prediction of NBGFR

The SRF-based model for predicting NBGFR utilized in this study was previously validated: Predicted NBGFR = 1.25 (Global GFR_Pre-RN_) (SRF_Contralateral_)^2,3^. SRF was derived in three different ways in each patient for comparison, one of which was by NRS. SRF was also obtained by differential PVA derived from (1) linear length–width-height (LWH) measurements; and (2) semi-automated software PVA analysis. Software-derived PVA- and LWH-based SRF of the contralateral kidney were calculated by normalizing the contralateral pre-RN parenchymal volume by the total pre-RN parenchymal volume (ipsilateral + contralateral). Contralateral RFC was estimated at 1.25, based on previous studies demonstrating an average of 20–30% compensatory hypertrophy in the 3–12 months post-RN^2,12,13^.

#### Software-derived PVA:

**Figure 2:**
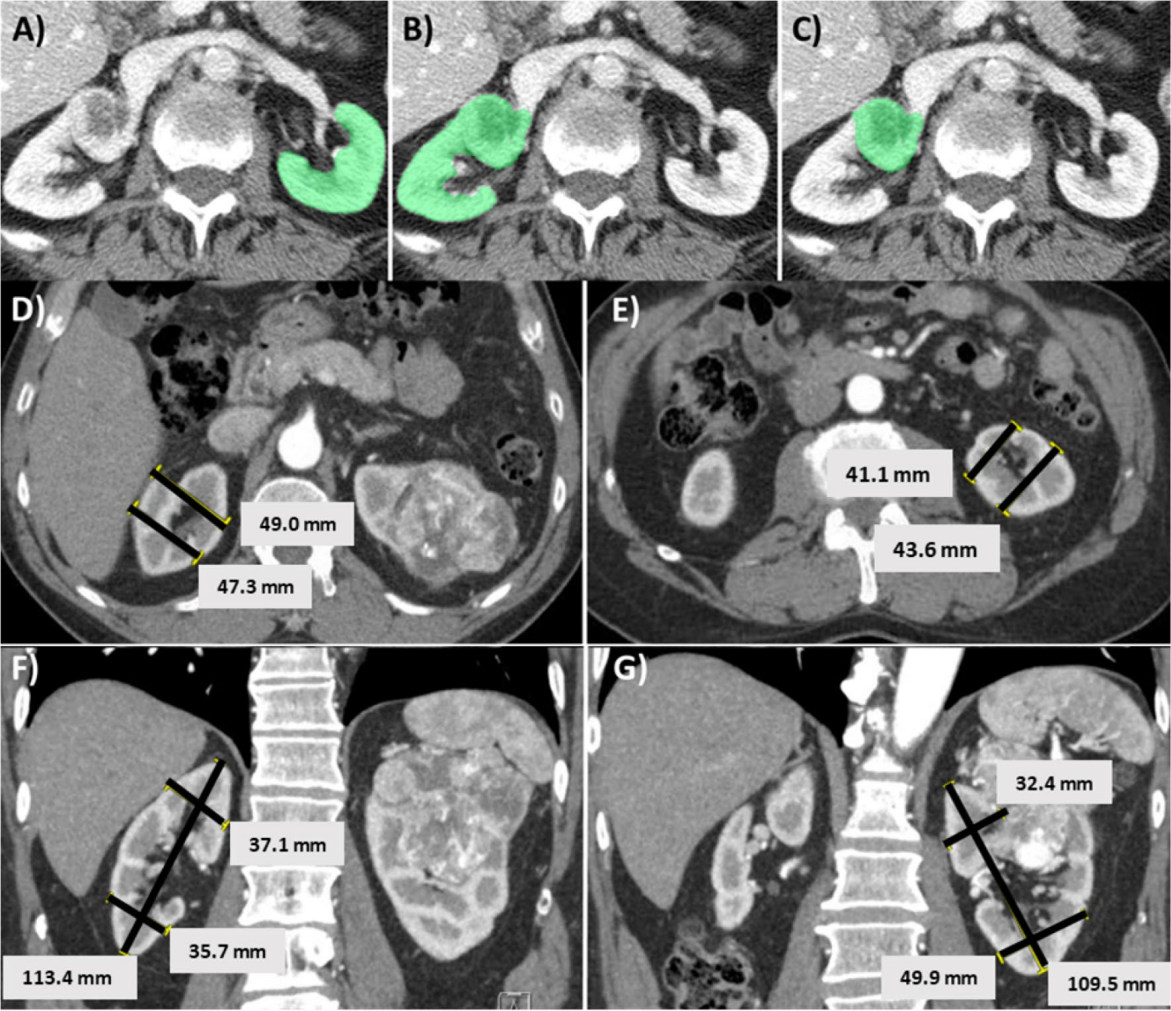
Software-derived parenchymal volume analysis (PVA) and linear length–width-height (LWH) measurements for calculating split renal function (SRF). (**A**–**C**) Software-based parenchymal volume analysis (PVA) of the (**A**) contralateral kidney (tumor-free); (**B**) ipsilateral kidney with tumor; and (**C**) tumor alone. In this case the left renal parenchymal volume was 210 cm^3^, the volume of the right renal parenchyma plus tumor was 229 cm^3^, and the tumor volume alone was 39 cm^3^. The parenchymal volumes were thus 210 cm^3^ (L) and 190 cm^3^ (R), and the estimated SRF was 53% (L) and 47% (R). (**D**,**E**,**F**,**G**) Linear length/width/height (LWH) measurements showing two anterior–posterior measurements (axial; **D**,**E**), and one length and two medial–lateral measurements (coronal; **F**, **G**) for each kidney. All measurements were obtained in cross-sectional images with maximal visible parenchyma, with exclusion of tumor and central sinus. Anterior–posterior and medial–lateral measurements were taken at the level of the polar line^15^. Parenchymal volumes were calculated as 0.52 × Length × (Average of anterior–posterior) × (Average of medial–lateral). In this example, the contralateral (tumor-free) kidney had a parenchymal volume of 0.52 × 11.3 cm (length) × 4.8 cm (anterior–posterior average) × 3.6 cm (medial–lateral average). The ipsilateral (tumor-bearing) kidney was 0.52 × 11 cm (length) × 4.2 cm (anterior–posterior average) × 4.1 cm (medial–lateral average). The relative parenchymal volumes were thus 98.5 cm^3^ (L) and 101.5 cm^3^ (R), and the estimated SRF was 49% (L) and 51% (R).

Preoperative parenchymal volumes of the ipsilateral (tumor-bearing) and contralateral (tumor-free) kidneys, and the tumor volume, were estimated from analyses of CT/MRI studies using a semi-automated 3D cross-sectional imaging software (Fujifilms Medical Systems) (Fig. 2A,B,C)^14^. Briefly, the software automatically extracts the renal parenchyma and/or tumor based on density features, and then the user can readily eliminate the renal cortex, vessels, sinus fat, and cysts if these are inadvertently included; which occurred in about 10–20% of the cases. Each case took approximately 3–5 min to obtain relevant parenchymal and tumor volumes, and thus SRF.

#### LWH-derived PVA:

Length, width, and height of the renal parenchyma were measured using the CT/MRI user interface of the electronic medical record. Briefly, two measurements in each of the anterior–posterior (Fig. 2D,E) and medial–lateral (Fig. 2F,G) dimensions, and one length measurement (Fig. 2F,G) were obtained, all adjusted for the natural axis of the kidney. All linear measurements were obtained from cross-sectional images that showed the maximal visible parenchyma, and excluded the tumor and the central sinus. Anterior–posterior measurements were taken from axial images at the polar line^15^. Medial–lateral measurements were taken from coronal images at the polar line. Length measurements were taken from the coronal image showing maximal length. Parenchymal volumes were calculated with the formula: 0.52 × length × (average of anterior–posterior measurements) × (average of medial–lateral measurements). Each case took, on average, 7–8 min to obtain parenchymal volumes and SRF. These analyses were performed by two co-authors (NR and KL). Twenty experimental, independent cases were evaluated by NR and KL to assess concordance, and the average discrepancy in SRF was 1.5% (range: 0–5%).

### Statistics

Continuous variables are reported as medians with interquartile ranges, and categorical variables as numbers with percentages. Predictive accuracies of the software-derived PVA, LWH, and NRS approaches were evaluated with Pearson correlation coefficients (*r*) and mean squared errors (MSE). The *r* values were determined by comparing the correlation between predicted and observed NBGFR for each method. MSE values quantify the discrepancy between predicted and observed NBGFR, with smaller MSE reflecting more accurate predictions of NBGFR. Additional performance parameters were also assessed for each approach, including bias, precision, and accuracy, as previously described^10^. Receiver operating characteristic (ROC) analyses were conducted to derive area under the curve (AUC) values that examined the ability of each model to discriminate postoperative NBGFR > 45 ml/min/1.73 m^2^. ROC analyses were performed only on the subset of patients who had a preoperative global GFR > 45 ml/min/1.73 m^2^ (n = 220). AUC, *r*, and additional performance parameters were compared across the three methods for deriving SRF using 1000 bootstrap resampling, with statistical significance set at *p* < 0.05 from two-sided tests. All analyses were performed using the statistical software R (r-project.org).

## Results

Patient characteristics and renal functional parameters for the final cohort of 235 RCC patients managed with RN who met all relevant inclusion criteria (Fig. 1) are shown in Table 1. Median age was 66 years and 69% of patients were male. Common comorbidities included hypertension (70%), cardiovascular disease (28%), diabetes (27%), and obesity, and 58% of patients were active or former tobacco users. Median Charlson-Comorbidity Index was 3. Median R.E.N.A.L. score was 10 and median tumor size was 5.9 cm. Surgical approaches were predominantly open (36%) or laparoscopic (44%). Pathologic tumor staging revealed pT3 or pT4 disease in 60% of patients, and histology was predominantly clear cell (70%). Patient clinical and demographic features and tumor characteristics were representative of RCC patients for whom RN is a relevant consideration in this era.

**Table 1:** Patient characteristics and renal functional parameters.

Patient demographics and tumor characteristics (n = 235)	
Median age (IQR)	66 (57–73)
Gender, N (%): Male/Female	161 (69)/74 (31)
Race, N (%): Caucasian/AA/Other	209 (89)/22 (9) / 4 (2)
Charlson comorbidity index, median (IQR)	3 (2–5)
BMI (kg/m^2^), median (IQR)	29.9 (26.9–35.1)
Diabetes, N (%)	64 (27)
Hypertension, N (%)	164 (70)
Cardiovascular disease, N (%)	65 (28)
Smoking, N (%): Never/Active or former	99 (42)/136 (58)
Tumor size (cm), median (IQR)	5.9 (4.5–8.2)
Preoperative CKD stage, N (%):	81 (34)
Stage 3, eGFR 30–60 ml/min/1.73 m^2^	78 (33)
Stage 4, eGFR 15–30 ml/min/1.73 m^2^	3 (1)
R.E.N.A.L. Score, median (IQR)*	10 (9–11)
Surgical approach, N (%)	
Laparoscopic/Robotic/Open	103 (44)/48 (20)/84 (36)
pT stage, N (%): pT1/pT2/pT3/pT4	72 (31)/22 (9)/137 (58)/4 (2)
pN stage, N (%): pN0/pN1/pNx	58 (25)/13 (5)/164 (70)
Histology, N (%)	
Clear cell/Papillary/Chromophobe	165 (70)/36 (15)/11 (5)
Other or unclassified	23 (10)
Renal function and parenchymal volume measurements	
*Preoperative*	
Function: Median preoperative eGFR (IQR)** Global Contralateral kidney Ipsilateral kidney (with malignancy)	70 (56–86) 39 (32–46) 30 (23–41)
Software-derived PVA measurements: Median (IQR) (cm^3^) Contralateral kidney parenchyma Ipsilateral kidney parenchyma (excluding tumor) Tumor alone	170 (134–210) 154 (121–190) 104 (37–246)
*Postoperative*	
Function: Median postoperative eGFR (IQR)** 3–12 months postoperative	47 (38–56)
Functional compensation***: Median % (IQR) 3–12 months postoperative	26 (1–41)

*AA* African–American, *BMI* body mass index; *CKD* chronic kidney disease; *eGFR* estimated glomerular filtration rate; *IQR* interquartile range; *R.E.N.A.L* (R)adius (tumor size as maximal diameter), (E)xophytic/endophytic properties of tumor, (N)earness of tumor deepest portion to collecting system or sinus, (A)nterior (a)/posterior (p) descriptor, and (L)ocation relative to polar lines.

*Includes data available from 189 patients.

**ml/min/1.73 m^2^.

***$$\frac{Postoperative\; eGFR - Preoperative\; contralateral \;eGFR}{{Preoperative \;contralateral \;eGFR}} \times 100$$.

Preoperative median global GFR was 70 ml/min/1.73 m^2^, with the ipsilateral and contralateral kidneys contributing a median of 30 ml/min/1.73 m^2^ and 39 ml/min/1.73 m^2^, respectively, based on SRF obtained from software-derived PVA (Table 1). Preoperative CKD was present in 34% of patients (33% grade 3 and 1% grade 4). Median ipsilateral and contralateral parenchymal volumes were 154 cm^3^ and 170 cm^3^, respectively, and the median tumor volume was 104 cm^3^. Median postoperative NBGFR was 47 ml/min/1.73 m^2^, with a median RFC of 1.26 (26% compensatory hypertrophy) observed in the contralateral kidney.

**Figure 3:**
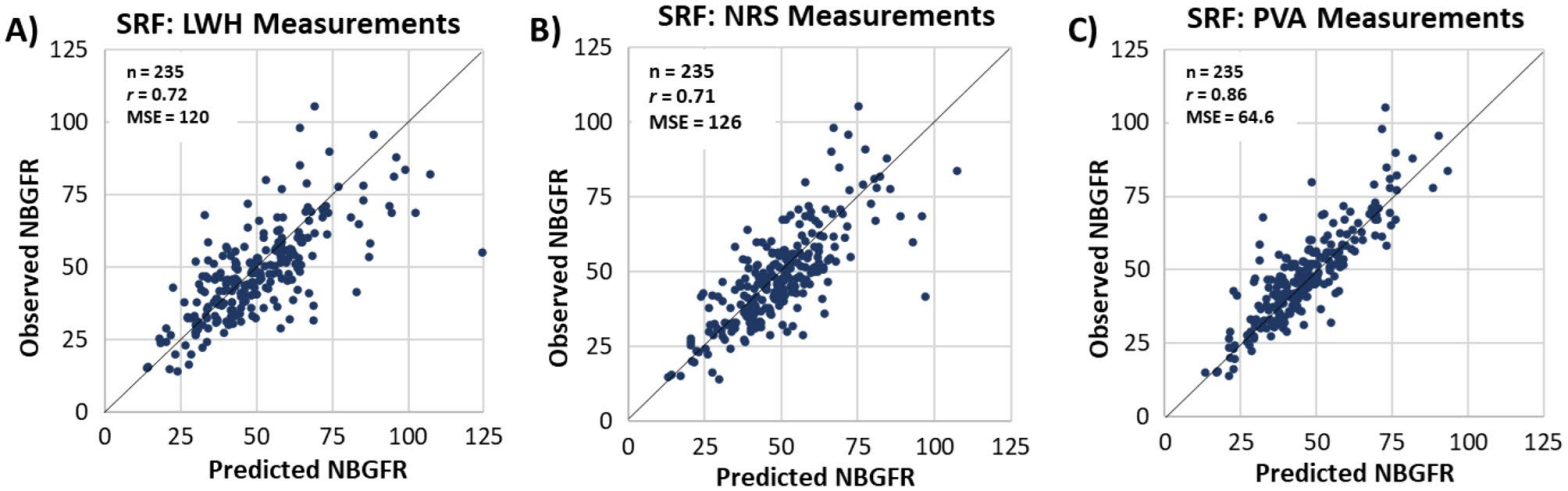
Correlation between predicted and observed new baseline GFR (NBGFR) after radical nephrectomy (RN). Comparison of observed and predicted NBGFR after RN based on split renal function (SRF) derived from **A**) Linear length/width/height (LWH) measurements, **B**) Nuclear renal scans (NRS), and **C**) Parenchymal volume analysis (PVA). The SRF-based model for obtaining predicted NBGFR was (1.25 × Global GFR_Pre-RN_ × SRF_contralateral_). Correlation coefficients (*r*) and mean-squared errors (MSE) that describe the predictive accuracies for each approach are provided.

Figure 3A,B,C show the correlations between predicted and observed NBGFR for the various methods for obtaining SRF. Correlation coefficients (*r*) were strong for the software-derived PVA approach (*r* = 0.86) and moderate for the LWH- and NRS-based approaches (*r* = 0.72 and 0.71, respectively). These differences were statistically significant (*p* < 0.03, Table 2). Software-derived PVA also demonstrated improved performance in terms of bias, precision, mean-squared error, and accuracy. Supplemental Figure 1 shows the ROC analyses that evaluate the abilities of each of the three methods in terms of discriminating postoperative NBGFR > 45 ml/min/1.73 m^2^. AUC values for the LWH, NRS, and software-derived PVA approaches were 0.72, 0.76, and 0.82, respectively.

**Table 2:** Performance of various approaches for estimating NBGFR after radical nephrectomy using distinct split renal function-based approaches.

	SRF derived from linear LWH measurements (*p* value)^Ϯ^	SRF derived from nuclear renal scan (NRS)	SRF derived from parenchymal volume analysis (PVA) (*p* value)^Ϯ^
Correlation (*r*)* [95% CI]	0.72 [0.64, 0.8] (0.012)	0.71 [0.6, 0.81] (0.023)	0.86 [0.81, 0.9]
Bias (ml/min/1.73 m^2^)** [95% CI]	− 2.75 [− 3.93, − 1.57] (0.011)	− 1.86 [− 3.06, − 0.5] (0.034)	0.44 [− 0.55, 1.49]
Precision^+^ [95% CI]	12.06 [9.97, 14.18] (0.03)	12.28 [9.91, 14.05] (0.032)	8.55 [7.07, 10.18]
Mean-squared error^++^ [95% CI]	120.41 [87.45, 151.6] (0.013)	126.05 [84.06, 166.02] (0.019)	64.55 [47.77, 82.76]
Accuracy (%)^ϒ^ [95% CI]	80.00 [74.89, 84.68] (0.004)	85.53 [81.28, 89.79] (0.059)	91.92 [88.09, 94.89]
AUC ^ϒϒ^ [95% CI]	0.72 [0.65, 0.78] (0.045)	0.76 [0.7, 0.82] (0.157)	0.82 [0.76, 0.87]

*AUC* area under curve; *CI* confidence interval; *NBGFR* new baseline glomerular filtration rate; *SRF* split renal function.

^+^Precision = interquartile range of bias (Q3 – Q1).

^ + +^ Mean-squared error = Average of the square of the error (difference between predicted and observed NBGFR): $\frac{1}{n} \times \sum_{i=1}^{n}{({Y}_{i}-{{Y}^{*}}_{i})}^{2}$ , where Y represents the model-predicted NBGFR and Y* represents the observed NBGFR.

^ϒ^Accuracy = percentage of predicted NBGFR values within 30% of observed NBGFR.

^ϒϒ^AUC values are obtained from ROC analyses that evaluate prediction of postoperative NBGFR > 45 ml/min/1.73 m^2^.

^Ϯ^*P* value is for comparison with SRF derived from PVA.

*Pearson correlation between predicted NBGFR and observed NBGFR.

**Bias = median of residuals (observed NBGFR – predicted NBGFR).

## Discussion

The 2021 American Urological Association (AUA) guidelines provide clear and granular recommendations for deciding between RN or PN for localized RCC patients^1^. These guidelines recommend that RN should be an important consideration for tumors with increased oncologic potential based on tumor size or concerning findings on renal mass biopsy or imaging^1^. In this setting, RN is preferred if (1) there is high tumor complexity that renders PN technically challenging or impractical, (2) there is no preexisting CKD or proteinuria, and 3) there is a healthy-appearing contralateral kidney that is likely to provide a NBGFR > 45 ml/min/1.73 m^2^ after RN^1^. The third criterion is of particular importance for challenging cases in which RN and PN each offer unique benefits and risks. As postoperative functional status can have significant implications for survival and quality of life, accurate prediction of NBGFR after RN can add valuable information to the clinical decision-making process^16^. Furthermore, accurate predictions of NBGFR can offer evidence-based expectations for postoperative care and prognosis for patients who must undergo RN for oncologic reasons despite functional concerns, which may be a strong possibility in real-world settings^17^.

Historically, predicting NBGFR after RN has been more challenging than after PN, with several complex, multivariate algorithms providing modest predictions at best^18–23^. Recent studies have shown that an intuitive model primarily based on SRF and RFC provides the most accurate predictions of NBGFR after RN, and can be readily implemented in the clinic due to its simplicity and practicality^2,3^. Post-RN RFC in adults reliably falls in the range of 1.20–1.30 (average 1.25) due to compensatory hypertrophy in the contralateral kidney^2,12,13^. SRF can be obtained in different ways, each with varying levels of accuracy and precision, yet the optimal method for estimating SRF remains unclear.

In this study, we compared three valid, quantitative techniques for estimating SRF and predicting NBGFR after RN. These approaches included NRS and differential PVA, with PVA obtained from either linear length/width/height (LWH) measurements or from software that performs automated kidney/tumor segmentations with an artificial intelligence-based algorithm. Our primary findings are that software-derived PVA provides the most accurate and precise estimations of SRF and predictions of NBGFR after RN, and that the LWH approach is noninferior to NRS with regards to these outcomes. Importantly, the PVA software is affordable, user-friendly, and available as an extension on standard electronic medical record interfaces, such as Epic Systems. These data support the implementation of two point-of-care PVA approaches that may obviate the need for NRS when evaluating functional outcomes after RN.

The traditional approach for estimating SRF has been NRS, which quantifies renal parenchymal function based on time-activity curves that are generated from a computerized assessment of radionuclide activity over the region of interest^4^. While NRS has the advantage of simultaneously delineating anatomy and function, there are several limitations. From a clinical perspective, hydration status, recent use of nonsteroidal anti-inflammatory drugs, and use of diuretic medications have been shown to reduce the accuracy of NRS^5^. From a technical standpoint, the orientation of the kidneys within the abdomen, impaired renal function, renal vascular thromboses, and the presence of renal cysts or perinephric collections can alter radiotracer uptake and/or excretion; thereby changing the extrarenal background activity^5^. Of note, these limitations are often present in patients with RCC, as they often have underlying comorbid conditions. Perhaps the most important shortcoming of NRS is an inherent subjectivity in defining the region of interest. Although the inter- and intra-observer variability in manual delineation of the region of interest is not unexpected, an automated image processing algorithm has been studied and did not successfully improve the detection of the region of interest^24^. In our study, the limited accuracy and notable variability of NRS in terms of evaluating SRF and NBGFR after RN are likely attributable to a combination of these factors.

We also present two methods for performing PVA to obtain SRF, both of which can be done at point-of-care. The first approach relies on linear LWH measurements that are obtained from axial and coronal views on CT/MRI. This technique is adapted from Feder et al. and Schober et al., who utilized such measurements to obtain differential renal parenchymal areas^8,9^. Our modified LWH approach, while similar in philosophy, has the advantage of quantifying a 3D anatomical structure with a volumetric measurement rather than with a 2D area measurement. PVA obtained from LWH measurements was generally straightforward, as each measurement could be made with simple tools present in electronic medical record. However, subjectivity is a concern with this approach. Despite the criteria for appropriate selection of cross-sectional images, there was a degree of inter-rater variability in terms of delineating the boundary at the tumor-parenchyma interface, particularly for endophytic tumors and those that invaded the central sinus, which are common scenarios for patients under consideration for RN. Additionally, obtaining accurate parenchymal measurements in the setting of multiple renal cysts is very time consuming, if not impractical. Despite these potential limitations, the LWH approach performed equally well in terms of estimating SRF and predicting NBGFR as compared to NRS. Our data from a robust sample size (n = 235) thus strengthens and validates the findings previously reported by Feder et al. (n = 111) and Schober et al. (n = 60). Since the LWH approach can be readily applied to routine CT/MRI studies, the need for NRS and the affiliated radiotracer exposure can arguably be forgone in most patients.

The second PVA technique relies on a software (FUJIFILM Medical Systems, USA) to extract anatomical structures of interest, in our case the renal parenchyma and tumor, from routine cross-sectional CT/MRI studies. Such medical imaging analysis programs apply deep learning algorithms that are trained to recognize anatomical signatures based on distinct visual and sequential features present on contrast-enhanced CT/MRI studies^25^. While the prospects of automated medical image analyses in clinical applications are promising, the potential technical limitations warrant a brief discussion. Image recognition algorithms require enormous training datasets, often on the magnitude of millions of diagnostic images^26^. In clinical settings, where such image analysis programs are often utilized for discrimination tasks in a disease-specific manner, datasets are much smaller, usually on the order of hundreds or thousands^26^. Additionally, gauging clinically-relevant predictions requires that the test data ideally match the actual target population, which may pose further restrictions^26^. These noteworthy obstacles create an inherent challenge in algorithm development and optimization.

Nevertheless, significant advances have been made in the application of deep learning-based computer vision in urology. The medical imaging software used in the present study has previously shown success in reconstructing 3D simulations of kidney-specific anatomy from CT/MRI, which has since helped facilitate clampless PN and predict residual renal function in kidney transplant settings^14,27^. The same software also led to more accurate estimations of NBGFR after RN in RCC patients when compared to subjective estimations of NBGFR made by expert urologic oncologists^6^. Recently, artificial intelligence algorithms have been developed to recognize distinct densities and morphological features of kidneys, kidney cysts, and kidney tumors on contrast-enhanced CT scans^28^. In polycystic kidney disease, such efforts have translated into very accurate automated estimations of total kidney volumes, which is virtually impossible using other methods^29^. In RCC, algorithms that perform automated kidney and tumor segmentations produce R.E.N.A.L. scores comparable to expert human-generated scores^30^. These algorithms accurately predicted oncological RCC outcomes, such as the presence of malignancy, necrosis, and high-grade and high-stage disease, among other relevant parameters^30^.

Our results further support the clinical utility of semi-automated, or even fully automated, kidney and tumor segmentation, specifically in terms of predicting functional outcomes for RCC interventions. The software used in our study readily provided reliable, objective parenchymal/tumor volumes from routine, contrast-enhanced CT scans, with only about 3–5 min of work per scan. This facilitated accurate and precise estimations of SRF, which in turn led to the most accurate predictions of NBGFR. We hypothesize that, in general, software-derived PVA overcame the clinical and technical limitations of NRS, and could more appropriately recognize complex tumors and cysts than the manual LWH method. However, software-based PVA did not accurately predict NBGFR for approximately 10–15% of patients, as defined by predicted NBGFR varying by at least ± 20% from the observed NBGFR. Future work is needed to identify the limitations of software-based PVA, specifically what features might distort the renal parenchyma volume/function relationship (e.g. hydronephrosis or renal scarring) or unique tumor phenotypes (e.g. infiltrative features), that may distort volumetric analyses. Nevertheless, it is evident that automated AI-generated segmentation models hold great promise in urological applications.

While the software-based PVA approach improved prediction of NBGFR relative to clinically-relevant threshold of 45 ml/min/1.73 m^2^, statistical significance was not met when compared to NRS (Supplementary Figure 1). A possible reason for this is the small sample size, which presents a potential limitation of this study. The sample size was restricted due to the need for NRS, which is not routinely obtained in this patient population. This raises additional considerations regarding the context of our study. Our study was single institutional and retrospective in design, which may also limit the generalizability of our findings. Nonetheless, our promising results highlight two point-of-care methods to evaluate SRF, and thereby accurately predict NBGFR after RN, in a facile and cost-effective manner that can be readily implemented in the clinic.

## Conclusion

We critically evaluated three distinct approaches to evaluate SRF and predict NBGFR after RN: NRS, linear LWH measurements, and a software-derived PVA. Software-derived PVA provides the most accurate and precise SRF estimations, and thus predictions of NBGFR after RN, when compared to NRS and LWH methods. Of note, the LWH method demonstrated equivalent performance as NRS in terms of predicting post-RN functional outcomes, precluding the need for NRS in most patients. Importantly, the software-derived PVA and LWH techniques can be performed in a facile manner at point-of-care, highlighting the strong potential for their clinical implementation. Accurate and precise predictions of NBGFR can inform clinical decision-making, particularly for challenging RCC cases in which RN and PN each have unique merits, and guide postoperative patient counseling in terms of survival and quality of life expectations when RN is imperative.

## Supplementary Information

Supplementary Information.

## Data Availability

The datasets used and/or analyzed during the current study available from the corresponding author on reasonable request.
