# Supplementary material for: Point of care parenchymal volume analyses to estimate split renal function and predict functional outcomes after radical nephrectomy
Source: Sci Rep. 2023 Apr 17;13:6225. doi: 10.1038/s41598-023-33236-6 (PMC10110585; doi:10.1038/s41598-023-33236-6)
Supplement: Supplementary file 1 — Supplementary Information. [file 41598_2023_33236_MOESM1_ESM.docx]

**Supplementary Figure 1**


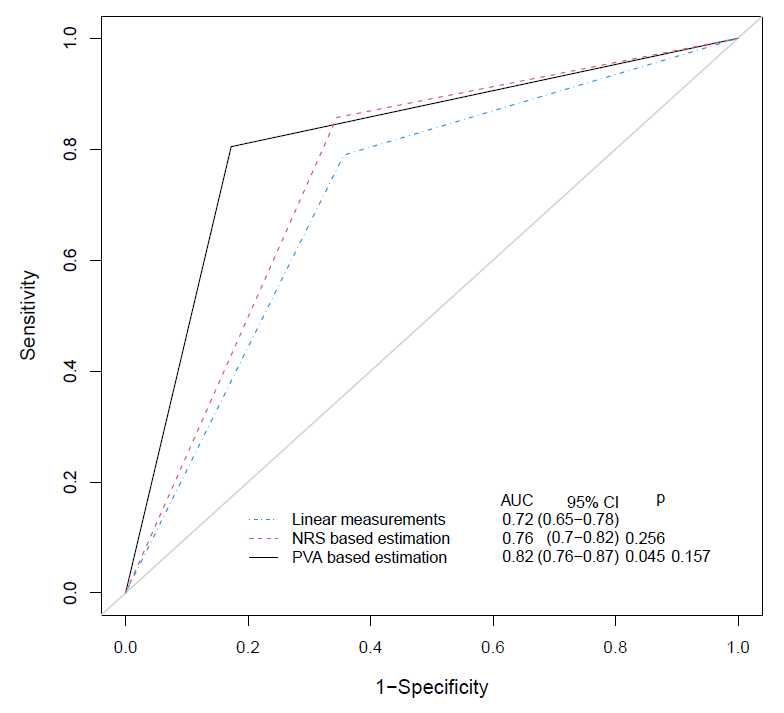


**Supplementary Figure 1: Receiver operating characteristics (ROC) curves that compare the abilities of linear length/width/height (LWH) measurements, nuclear renal scans (NRS), and parenchymal volume analysis (PVA) to discriminate postoperative NBGFR > 45 ml/min/1.73m^2^.** The area under the curves (AUC) are based on characterization of the postoperative NBGFR relative to the 45 ml/min/1.73m^2^ threshold as a binary variable.
